# Vitrectomy with Inverted Fovea-Sparing Internal Limiting Membrane for Myopic Foveoschisis

**DOI:** 10.1155/2022/3242747

**Published:** 2022-02-12

**Authors:** Xinsheng Li, Jingfan Wang, Yan Wu, Xingxing Wang, Zhengyu Zhang, Zizhong Hu, Ping Xie

**Affiliations:** Department of Ophthalmology, The First Affiliated Hospital of Nanjing Medical University, Nanjing, Jiangsu 210029, China

## Abstract

**Purpose:**

To evaluate the efficacy and safety of vitrectomy with inverted fovea-sparing internal limiting membrane, as a modified surgical technique in the treatment of the eyes with myopic foveoschisis.

**Methods:**

This study was based on a consecutive, interventional case series. A standard 25-gauge (25-G), 3‐port pars plana vitrectomy combined with inverted fovea-sparing internal limiting membrane was performed on 13 eyes. Preoperative and postoperative best-corrected visual acuity, optical coherence tomography image, and central foveal thickness were analyzed. Patients were followed up for at least 6 months.

**Results:**

All 13 eyes showed dramatical resolution of myopic foveoschisis during the follow-up. The mean logarithm of minimum angle of resolution best-corrected visual acuity showed remarkable improvement from 1.06 ± 0.42 to 0.45 ± 0.25 (*p* < 0.0001; paired *t*-test). The mean central foveal thickness significantly decreased from 479.62 ± 113.16 *μ*m to 372.38 ± 88.12 *μ*m, 316.18 ± 73.97 *μ*m, and 272.40 ± 61.32 *μ*m postoperatively at 1 month, 3 months, and 6 months, respectively (*p* < 0.0001; paired *t*-test; preoperation vs. latest follow-up).

**Conclusions:**

Vitrectomy with inverted fovea-sparing internal limiting membrane can resolve myopic foveoschisis with high efficacy and safety.

## 1. Introduction

Pathological myopia was originally described as high myopia (refractive error of –6 diopters (D) or more) accompanied by characteristic degenerative changes in the retina, choroid, and sclera [1]. Myopia foveoschisis (MF), also known as myopic traction maculopathy, is a common pathological myopic complication that leads to a gradual and painless diminution of vision. Among the 2% of the world population suffering from pathological myopia [2], MF accounts for 8–34% [3]. MF on optical coherence tomography (OCT) usually manifests the splitting of the inner retinal layer with column-like formations [4].

The exact pathogenesis of MF is still poorly understood, and the tractional forces on the retina may be the primary cause that leads to the separation of retinal neuroepithelial layer [5]. Other etiologies may include premacular cortical vitreous, stiff retinal vasculature, internal limiting membrane (ILM) rigidity, and posterior staphyloma [6]. Currently, vitrectomy combined with fovea-sparing ILM peeling is a recognized option for the management of MF. However, it is a great challenge to retain the foveal ILM in a way that is more convenient and minimizes the damage to the fovea for eye surgeons.

Over the past decade, diverse surgical techniques have been introduced for fovea-sparing ILM peeling [7–11], and these techniques require intense training and longer learning curve to be mastered, especially for young surgeons. Here, we report a reliable but relatively simple technique for the management of MF.

## 2. Materials and Methods

This study was based on a consecutive, interventional case series performed in the First Affiliated Hospital of Nanjing Medical University. Thirteen eyes of 13 patients were included from June 2020 to September 2021. Inclusion criteria: myopic refractive error higher than −6.00 D and axial length (AL) longer than 26 mm; presence of foveoschisis scanned by spectral-domain (SD)-OCT (Cirrus HD-OCT; Carl Zeiss Meditec Inc., Dublin, CA). Exclusion criteria: ocular disorders like myopic choroidal neovascularization, full thickness macular hole, ocular trauma, diabetic retinopathy, and age-related macular degeneration. All patients underwent detailed examinations about intraocular pressure, best-corrected visual acuity (BCVA), and fundus. BCVA was recorded in Snellen charts and converted to a logarithm of minimum angle of resolution (logMAR) for statistical analysis. Patients were followed up for at least 6 months.

### 2.1. Surgical Technique

**Figure 1:**
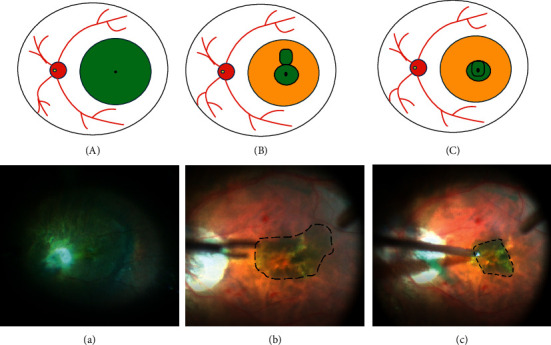
Schematic drawings and surgical video screenshots. ILM stained with indocyanine green ((a), (A)). Foveal ILM with a flag retained during peeling off ILM ((b), (B)). The flag inverted to structure double-layer ILM on the surface of the fovea ((c), (C)).

The steps in inverted fovea-sparing ILM peeling are shown in Figure 1. Anesthesia was implemented through the retrobulbar nerve block. A standard 25-gauge (25-G), 3-port pars plana vitrectomy was performed by an experienced surgeon (P.X) using the vitrectomy system (Alcon Laboratories Inc., FortWorth, TX) and noncontact (wide-angle) viewing system Resight 700 (Carl Zeiss Meditec AG, Jena, Germany). The main surgical procedure is as follows:

1. Unless it has already spontaneously occurred, a posterior vitreous detachment is induced. The central core vitreum and posterior hyaloid were thoroughly removed with the assistance of triamcinolone.
2. The ILM was visualized through staining with 0.1 mL of indocyanine green (ICG, 1.25 mg/mL, Eisai, Inc. Shenyang, China) for 30 s, followed by an immediate lavage
3. The peripheral area of the macula was first peeled and then extended to the edge of the posterior scleral staphyloma and upper-lower vessel arcs. Foveal ILM and a tongue-shaped flag were retained without excessive trimming.
4. The edge of ILM flap is gently inverted to cover the fovea using microforceps
5. Air-fluid exchange is performed using a 23/25-gauge flute needle
6. Finally, the vitreous cavity is filled with silicon oil. The patients were suggested to maintain a prone position for at least 1 week and lateral position for 2 weeks postoperatively.

### 2.2. Statistical Analysis

SPSS software version 20.0 (SPSS Inc., Chicago, IL) was used to perform statistical analyses of the data. Preoperative and postoperative central foveal thickness (CFT) and LogMAR BCVA were compared by paired *t*-tests. *P* value <0.05 was considered statistically significant.

## 3. Results

This modified surgical technique has been applied in 13 eyes. The preoperative demographics of 13 patients are given in Table 1. The patient population consisted of 10 male (77.8%) and 3 female, with a mean age of 55.23 ± 13.56 years (range: 35–74 years). The mean AL was 29.52 ± 1.81 mm (range: 27.50–32.55 mm). The mean preoperative BCVA was 1.06 ± 0.42 (range: 0.3–1.6). The mean CFT was 479.62 ± 113.16 *μ*m (range: 550–302 *μ*m). The mean follow-up was 7.38 ± 2.84 months.

**Figure 2:**
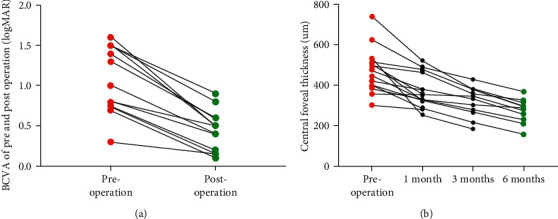
Pre and postoperative visual outcomes and central foveal thickness. The mean logMAR BCVA increased from 1.06 ± 0.42 to 0.45 ± 0.25 (a). The CFT decreased from 479.62 ± 113.16 *μ*m to 372.38 ± 88.12 *μ*m, 316.18 ± 73.97 *μ*m, and 272.40 ± 61.32 *μ*m postoperatively at 1 month, 3 months, and 6 months, respectively (b).

After surgical intervention, the mean logMAR BCVA remarkably increased from 1.06 ± 0.42 to 0.45 ± 0.25 (*p* < 0.0001; paired *t*-test) (Figure 2(a)**)**. Meanwhile, the central foveal thickness dramatically decreased from 479.62 ± 113.16 *μ*m to 372.38 ± 88.12 *μ*m, 316.18 ± 73.97 *μ*m and 272.40 ± 61.32 *μ*m postoperatively at 1 month, 3 months, and 6 months, respectively (*p* < 0.0001; paired *t*-test; preoperation vs. latest follow-up) (Figure 2(b)). All the 13 cases recovered smoothly without intraoperative or postoperative complications and the formation of macular holes.

**Figure 3:**
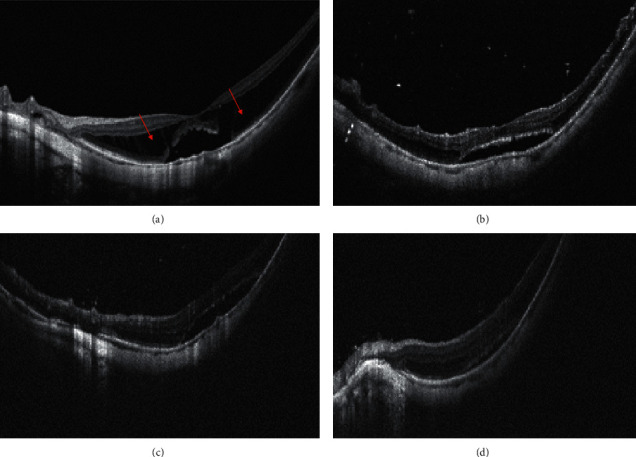
Pre and postoperative representative OCT images of simple myopic foveoschisis. Preoperative image shows the splitting of inner retinal layer with column-like formations (red arrow), and the CFT and BCVA were 493 *μ*m and 1.5, respectively (a). At 1 month after operation, the CFT was 466 *μ*m (b). At 3 months after operation, the CFT was 359 *μ*m (c). At 6 months after operation, the CFT and BCVA were 324 *μ*m and 0.5, respectively (d).

**Figure 4:**
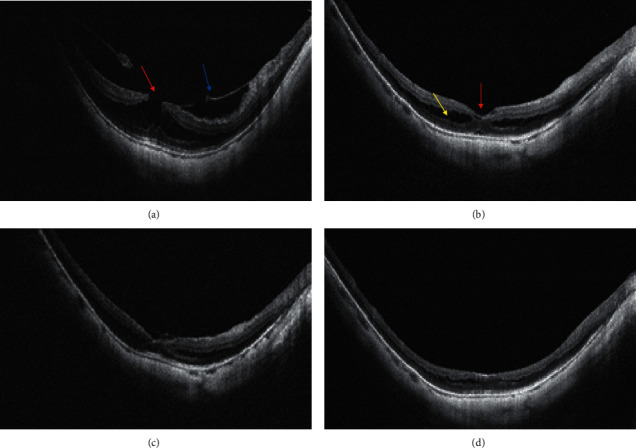
Pre and postoperative representative OCT images of myopic foveoschisis accompanied by the lamellar macular hole. Preoperative image shows a foveoschisis, lamellar macular hole (red arrow), and posterior vitreous detachment (blue arrow). The CFT and BCVA were 505 *μ*m and 0.7, respectively (a). At 1 month after operation, the foveal schisis was fully reattached (red arrow), but parafoveal schisis still remained (yellow arrow). The CFT was 323 *μ*m (b). At 3 months after operation, the parafoveal schisis was gradually resolved, and the CFT was 304 *μ*m (c). At six months after operation, parafoveal schisis and foveoschisis were completely resolved, and the CFT and BCVA were 290 *μ*m and 0.15, respectively (d).

Figure 3 shows pre and postoperative OCT images of simple MH (case 3). Preoperative OCT demonstrated the splitting of inner retinal layer with column-like formations. Six months after operation, foveoschisis almost reattached, CFT decreased from 493 *μ*m to 324 *μ*m, and BCVA improved from 1.5 to 0.5. In addition, the representative OCT images of the MF eye accompanied by a lamellar macular hole (case 1) is shown in Figure 4. The preoperative OCT image showed the presence of foveoschisis with a lamellar macular hole and posterior vitreous detachment. One month after operation, foveal schisis was fully reattached, but parafoveal schisis still remained. At six months reexamination, parafoveal schisis was also resolved, CFT decreased from 505 *μ*m to 290 *μ*m, and BCVA improved from 0.7 to 0.15.

## 4. Discussion

This modified technique could repair foveoschisis and improve visual acuity. During the follow-up, no macular holes and other severe complications were observed. Vitrectomy has been considered as therapeutic for disorders in the ocular posterior segment [12]. The ILM, a basement membrane composed of Müller cells, is located at the border between the vitreum and retinal neuroepithelium. It is critical for maintaining homeostasis of the eye, and its pathological changes may contribute to a variety of vitreoretinal diseases [13]. Vitrectomy combined with ILM peeling is a widely performed technique procedure performed by vitreoretinal surgeons for myopic traction maculopathy. However, there is still a debate regarding the suitability of complete macular ILM peeling or fovea-sparing ILM peeling to high myopic foveoschisis. In a retrospective, observational study, Iwasaki et al. reported that a full-thickness macular hole did not develop after fovea-sparing ILM peeling, but in 27.3% (3/11) of the eyes after complete ILM peeling [14]. Shimada et al. also reported similar outcomes [9]. Complete foveal ILM peeling may damage the basement membrane of the Müller cells and anatomical structure of fovea, leading to postoperative adverse events, such as macular hole, even macular hole retinal detachment. Therefore, we believe that fovea-sparing ILM peeling is more preferable for the eyes with myopic traction maculopathy.

Recently, diverse techniques have been invented to preserve foveal ILM. Jin et al. described a parafoveal multiple curvelinear ILM peeling technique [7]. In their surgery, ILM was peeled off from the central fovea in a continuous curvilinear manner in each quadrant. After the parafoveal curvilinear ILM peeling, small areas of residual ILM between the four circular areas of ILM were removed. “Continuous Arc-Shaped Foldback Peeling” and “maculorrhexis” techniques using microscissors or a vitreous cutter have been reported to trim the irregular edge of retained ILM [10, 11]. Since the ILM in high myopia eyes is thin and fragile [8], challenges remain in previous techniques in retaining foveal like-circular ILM. On the one hand, the stripped ILM is easily broken, and the direction of free ILM edge cannot be well controlled during stripping. When surgeons trim the retained ILM, the retina faces a risk of being injured by the tip of microscissors. Moreover, the vitreous cutter may suck away the retained ILM and injure the retina under poorly controlled negative pressure.

As shown in Figures 3 and 4, for cases with extremely thin retinal neuroepithelium or with lamellar macular holes, full-thickness macular holes and retinal detachment are highly prone to develop. We hope inverted double-layer foveal ILM can not only facilitate foveoschisis reattachment but also prevent the development of macular holes. Outcomes of the two cases were satisfactory, without any surgical complications at the latest follow-up, and their visual acuity and central foveal thickness improved significantly.

Nevertheless, this study has several limitations. First, we only performed this technique in 13 cases. The sample size was relatively small, and the follow-up period should be prolongated. Second, we did not perform a cross-sectional study to compare the outcomes of fovea-sparing ILM peeling and inverted ILM technique. Finally, more indicators, such as microperimetry and multifocal electroretinogram, should be utilized to comprehensively evaluate postoperative visual function.

## 5. Conclusions

In conclusion, vitrectomy combined with inverted fovea-sparing ILM can be applied for myopic foveoschisis, even concurrent with lamellar macular holes. More clinical studies should be conducted before its wide replication.

## Figures and Tables

**Table 1:** Preoperative demographic.

Case	Age/gender/eye	AL	BCVA (logMAR)	CFT	Follow-up (months)
1	69/F/R	31.15	0.7	505	9
2	49/F/L	28.07	0.7	357	10
3	65/F/L	29.96	1.5	493	8
4	74/F/L	29.03	1.0	624	7
5	69/F/R	32.38	1.6	739	10
6	69/F/R	28.79	0.73	511	11
7	38/M/L	30.68	1.3	385	7
8	51/M/R	32.55	1.4	530	10
9	39/F/R	27.57	0.3	302	2
10	51/F/R	28.10	0.8	421	7
11	63/F/L	30.40	1.5	445	6
12	46/F/L	27.52	1.5	448	7
13	35/M/R	27.50	0.8	475	2

F, female; M, male; R, right eye; L, left eye; AL, axial length (mm); BCVA, best-corrected visual acuity; logMAR, logarithm of minimum angle of resolution; CFT, central foveal thickness (*μ*m).

## Data Availability

The datasets used and/or analyzed during the current study are available from the corresponding author upon request.
